# Exploring the DPP-IV Inhibitory, Antioxidant and Antibacterial Potential of Ovine “Scotta” Hydrolysates

**DOI:** 10.3390/foods10123137

**Published:** 2021-12-17

**Authors:** Roberto Cabizza, Francesco Fancello, Giacomo Luigi Petretto, Roberta Addis, Salvatore Pisanu, Daniela Pagnozzi, Antonio Piga, Pietro Paolo Urgeghe

**Affiliations:** 1 Dipartimento di Agraria, Università degli Studi di Sassari, Viale Italia 39/A, 07100 Sassari, Italy; rcabizza@uniss.it (R.C.); fancello@uniss.it (F.F.); pigaa@uniss.it (A.P.); 2 Dipartimento di Chimica e Farmacia, Università degli Studi di Sassari, Via Muroni 23/A, 07100 Sassari, Italy; gpetretto@uniss.it (G.L.P.); raddis@uniss.it (R.A.); 3 Porto Conte Ricerche SRL, Loc Tramariglio, 07041 Alghero, Italy; pisanu@portocontericerche.it (S.P.); pagnozzi@portocontericerche.it (D.P.)

**Keywords:** ovine scotta, bioactive peptides, bromelain, pancreatin, dipeptidyl peptidase IV inhibition, ovine second whey cheese, enzymatic hydrolysis

## Abstract

The aim of this work was to valorize the by-product derived from the ricotta cheese process (scotta). In this study, ovine scotta was concentrated by ultrafiltration and then subjected to enzymatic hydrolyses using proteases of both vegetable (4% E:S, 4 h, 50 °C) and animal origin (4% E:S, 4 h, 40 °C). The DPP-IV inhibitory, antioxidant, and antibacterial activities of hydrolysates from bromelain (BSPH) and pancreatin (PSPH) were measured in vitro. Both the obtained hydrolysates showed a significantly higher DPP-IV inhibitory activity compared to the control. In particular, BSPH proved to be more effective than PSPH (IC_50_ 8.5 ± 0.2 vs. 13 ± 1 mg mL^−1^). Moreover, BSPH showed the best antioxidant power, while PSPH was more able to produce low-MW peptides. BSPH and PSPH hydrolysates showed a variable but slightly inhibitory effect depending on the species or strain of bacteria tested. BSPH and PSPH samples were separated by gel permeation chromatography (GPC). LC-MS/MS analysis of selected GPC fractions allowed identification of differential peptides. Among the peptides 388 were more abundant in BSPH than in the CTRL groups, 667 were more abundant in the PSPH group compared to CTRL, and 97 and 75 of them contained sequences with a reported biological activity, respectively.

## 1. Introduction

“Scotta”, also called “ricotta cheese exhaust whey” (RCEW) or “second cheese whey” (SCW) is the residual liquid by-product of the ricotta cheese production process, obtained by thermal flocculation of whey proteins, and heating the whey at a temperature of 85–90 °C and 78–85 °C for bovine or buffalo and ovine or goat whey, respectively [1]. Whey composition, the treatments performed during ricotta production process (i.e., adding milk, whey protein extraction method), and ricotta yield (depending on the temperature of protein coagulation, pH, and ionic strength of the whey) are the main factors that affect the physicochemical characteristics of scotta [2].

In Italy, scotta is mainly produced from bovine and ovine milk and, in less quantity, from buffalo and goat milk. During 2019 about 900,000 tons [3] of whey were transformed into ricotta cheese in Italy, representing about 16% of total whey produced, giving rise to more than 750,000 tons of scotta.

The production of scotta from ovine whey is mainly concentrated in Sardinia, which hosts about 22% of Italian dairy ewes [4] and where is produced more than 65% of Italian ovine milk. The Sardinian dairy system produces about 320,000 tons of ovine milk [5], which is almost completely destined for cheese production. About 12,000 tons of ricotta are produced in Sardinia, with a potential production of more than 250,000 tons of scotta. The disposal of scotta poses serious environmental concerns due to its high biochemical oxygen (BOD) and chemical oxygen demand (COD) [6,7,8]. Therefore, its valorization is becoming crucial for the dairy industry.

For many decades whey, and especially scotta, were underused or treated as waste, due to poor knowledge of their valuable components and the unavailability of adequate technologies to valorize such components. Over the years, several approaches to its valorization have been developed [9,10,11], since scotta still retains significant amount of useful compounds from whey, such as lactose, minerals, oligosaccharides, vitamins, proteins, soluble peptides, and free amino acids [9]. Scotta has been employed using biotechnological approaches as a growth substrate for some selected Lactic Acid Bacteria (LAB) for the production of lactic acid [12], or yeasts such as *Chlorella protothecoides* for the production of carotenoids, chlorophyll [13], and bioethanol [6,14], as ingredient to fortify ricotta, including bioactive peptides with antioxidant and anti-tyrosinase activities [15], bioactive peptides with angiotensin-I-converting enzyme (ACE)—inhibitory [16] biodegradable bioplastic [17], fermented drink [18] and hydrogen [19,20].

Furthermore, the interest in the application of enzymes of animals or plant sources applied to food matrices has grown during over the years [21,22], with the aim of valorizing by-products and reducing environmental impact [23]. The hydrolysis of whey proteins is a widespread practice, and the hydrolyzed whey protein (WPH) has had an important impact as functional or nutraceutical ingredient. WPH is produced by enzymatic hydrolysis of whey proteins, mainly from whey protein concentrates (WPC) or isolates (WPI), which leads to an increase in solubility and digestibility, reducing whey protein allergenic properties. Peptides obtained by enzymatic hydrolysis of whey proteins have shown interesting biological activities, with potential benefit for human health [24,25]; however, scientific studies specifically addressed to the valorization of scotta proteins and derived peptides are still poor [15].

Recently, several authors studied the potential of whey protein hydrolysates as inhibitors against dipeptidyl peptidase-4 (DPP-IV) [26,27,28,29]. DPPI-IV inhibitors play a key role in the treatments of type 2 diabetes (T2D), a worldwide diffused disease that affects 415 million people and in Italy accounts for more than 3 million patients, i.e., about 5% of the population [30].

The DPP-IV inhibitors adopted as oral antidiabetic agents act by promoting glucose homeostasis through the inhibition of the enzyme DPP-IV involved in the mechanism of degradation of glucose-dependent insulinotropic polypeptide (GIP) and glucagon-like peptide-1 (GLP-1), two key glucoregulatory hormones. DPP-IV inhibitors can reduce glucagon levels and at the same time stimulate insulin release.

The antioxidant potential of peptides derived from milk and whey proteins is well-reviewed [31]. However, up to now, just two works have focused on the antioxidant activity of scotta. Sommella et al. [11] reported bovine scotta peptides with antioxidant activity derived from αs1-casein and β-casein. Monari et al. [15] tested different proteases on bovine scotta, observing in vitro the antioxidant potential of the obtained hydrolysates.

The antibacterial activity of biopeptides encrypted into the protein fraction of ovine milk and whey has been already shown [32,33,34]. Several peptides of the C-terminal region of the ovine αs2-casein were shown to possess antibacterial activity against Gram-positive and Gram-negative bacteria, with the first group of bacteria more susceptible than the second group [33]. El-Zahar et al. [32] found that the peptic hydrolysates of whey protein inhibited, in a dose-dependent manner, the growth of *Escherichia coli*, *Bacillus subtilis*, and *Staphylococcus aureus*. Regarding scotta, recent work [11,15] showed the presence of several antibacterial peptides in the protein fraction of scotta.

Regarding the available proteases, bromelain and pancreatin were previously applied in the hydrolysis of dairy products and by-products to produce peptides with DPP-IV inhibitory [35] and antioxidant activity [15]. In addition, bromelain was reported to release antibacterial peptides from goat milk and whey [36].

While the literature has been mainly focused on the bioactive properties of peptides obtained from milk and whey proteins, the available data on scotta are limited to the bovine source [9] and, to the best of our knowledge, the present study is the first conducted on the ovine scotta as a potential source of bioactive peptides. Conversely, this matrix could represent an important source of such peptides, due to its higher amount of nitrogen, compared with bovine whey and scotta. The research conducted in this field could lead both to an adequate valorization of this by-product and an improvement of the sustainability of the dairy farms. Therefore, the aim of the present study was to evaluate the possibility of producing peptides with DPP-IV inhibitory, antioxidant and antibacterial activities from ultrafiltered ovine scotta by enzymatic hydrolysis with bromelain (BSPH), and pancreatin (PSPH), and evaluate their biological activity by in vitro tests. Furthermore, an in-depth characterization of peptide mixtures obtained from the hydrolysis with the two enzymes by gel permeation chromatography (GPC) and LC-MS/MS was performed.

## 2. Materials and Methods

### 2.1. Chemicals and Reagents

Analytical grade chemicals were obtained from Carlo Erba (Milano, Italy). Bromelain was obtained from Nutraceutica (Bologna, Italy) (2400 GDU g^−1^) and from Enzyme Development Corporation (New York, NY, USA). Pancreatin from porcine pancreas (4 USP) was purchased from Sigma-Aldrich (St. Louis, MI, USA).

Synthetic peptides used for Gel Permeation Chromatography (GPC) calibration were bought from Sigma-Aldrich (St. Louis, MI, USA) (i.e., bovine serum albumin (BSA), β-Lactoglobulin (β-Lg), α-lactalbumin (α-La), aprotinin, bacitracin, tetrapeptide (Leu-Trp-Met-Arg), Asp-Glu, Tyr).

### 2.2. Scotta Concentrate Preparation

Ovine scotta was freshly collected from a dairy plant located in north Sardinia (Italy) after the manufacturing of ovine Ricotta cheese, and immediately refrigerated to 4 °C, delivered to the lab and stored at −20 °C. Scotta was thawed to 20 °C immediately before the concentration step. The mean chemical composition, determined according to the literature [37] was as follows: pH 6.19 ± 0.11; total solids, 6.73 ± 0.28% (*w*/*w*); fat, 0.05 ± 0.02% (*w*/*w*); total nitrogen (TN), 0.14 ± 0.01% (*w*/*w*); nitrogen soluble in water (NS), 0.08 ± 0.05% (*w*/*w*); non-protein nitrogen (NPN), 0.07 ± 0.05% (*w*/*w*); ash, 0.34 ± 0.23% (*w*/*w*).

Scotta (5 L) underwent a preliminary continuous skimming at 15,000× *g* using a lab-scale cream separator (TLE 100, Tecnolatte, Lodi, Italy), then was consecutively filtered through 5, 1.2, and 0.65 µm on conventional cartridge filters (Sartopure PP3 Midicap, Sartorius, Goettingen, Germany) with a surface area of 0.21, 0.15, and 0.15 m^2^ respectively, fed by a SartoJet Membrane Pump (Sartorius, Goettingen, Germany). The 0.65 µm filtered skimmed scotta underwent tangential filtration at 20 °C through a 10 kDa Hydrosart membrane (Sartocon Slice Cassette, Sartorius, Goettingen, Germany), keeping a constant transmembrane pressure of 0.5 bar, and monitoring the removed permeate weight, which was precalculated in order to obtain a nitrogen concentration factor of 4×. The retentate was then reconstituted to the original weight by adding ultrapure (UP) water and subsequently underwent a diafiltration step using the same membrane, in order to reduce the mineral fraction and lactose. The collected retentate was sampled at the end of the process for total nitrogen (NT) determination and then stored at −20 °C until the subsequent hydrolysis steps.

### 2.3. Enzymatic Hydrolysis of Retentate Scotta Samples

The retentate obtained in Section 2.2, with a total nitrogen of 0.50 ± 0.01% (*w*/*w*), was split between two experiments to be hydrolyzed with bromelain (BSPH) and pancreatin (PSPH), respectively. For each experiment, three hydrolyses on 50 g (*n* = 3) of retentate were performed. Lab-scale enzymatic reactions were performed for 4 h. Enzymatic hydrolysis were performed, as recommended by the manufacturer, at 50 °C for bromelain and at 40 °C for pancreatin. The enzyme-substrate (E:S) ratio was fixed at 4% (enzyme weight to protein weight) for both experimental groups. Further three 50 g retentate control samples (CTRL) underwent the same procedure without enzyme addition, setting the temperature and duration to 50 °C and 4 h, respectively. The enzymatic reactions were performed in a water bath at constant temperature (±0.05 °C) and continuous magnetic stirring at 500 rpm using an AREX-6 Digital PRO Hot Plate Stirrer (Velp Scientific, Bohemia, NY, USA) equipped with a VTF EVO digital thermoregulator. All the reactions were stopped by heating the mixtures at 90 °C for 10 min to inactivate the proteases. Afterward, the mixtures were centrifuged twice at 14,000× *g* (Neya 16 R, Remi Elektrotechnki LTD, Vasai, India) for 15 min at 4 °C, and the precipitate was discarded. The obtained supernatants of bromelain scotta protein hydrolysate (BSPH), pancreatin scotta protein hydrolysate (PSPH) and of the control were freeze-dried (Labconco, Kansas City, MO, USA) and stored at −20 °C until further analysis, such as DPP-IV inhibition, antioxidant capacity and an antibacterial assay, as well as gel permeation chromatography (GPC) and LC-MS/MS characterization.

### 2.4. DPP-IV Inhibitory Activity

A DPP-IV drug discovery kit was used to measure the ability of hydrolysates to inhibit DPP-IV activity (Enzo Life Sciences Inc., Farmingdale, New York, NY, USA). The assays were conducted according to the manufacturer’s instructions. Briefly, the kit contained human recombinant DPP-IV enzyme, a chromogenic substrate (H-Gly-Pro-pNA, MW = 328.8, 10 mM in DMSO), a calibration standard (p-nitroaniline, MW = 138, in assay buffer), an inhibitor as positive control (P32/98, MW = 260.4, 1 mM in DMSO), and an assay buffer (50 mM Tris, pH 7.5). All the reagents of the kit were stored at −70 °C; the analyses were conducted at room temperature. The freeze-dried protein hydrolysates were dispersed in ultrapure water in concentrations from 0.78 to 12.5 mg mL^−1^. Assays were performed at 37 °C, in a 96-well microplate provided by the manufacturer and the reading was performed in a microplate reader every minute for a total of 30 min at λ 405 nm. Finally, absorbance values were plotted against time, and the “best fit” lines for data points and slope of the curves were obtained. Two technical replicates for each sample were performed. The % of inhibition was calculated with the formula:

% activity remaining (with inhibitor) = (slope of inhibitor sample/control slope) × 100

The obtained data were analyzed by a one-way analysis of variance (ANOVA) using a Statgraphics Centurion XVI for Windows software package (version 16.2.04; Statpoint Technologies, Inc. Warrenton, Virginia, VA, USA). Fisher’s least significant differences (LSD) test was applied to assess the difference between each pair of means (*p* < 0.05).

### 2.5. ABTS Radical Scavenging Activity

Antioxidant capacity was evaluated by colorimetric assay measuring the activity of the sample to scavenge the radical ABTS according to the method described by Petretto et al. [38]. The ABTS radical scavenging activity is based on the production of the radical cation (ABTS·+), prepared by reacting ABTS and potassium persulfate (2.45 mM) to reach a final concentration of 7 mM. Briefly, the solution obtained was kept in the dark at 25 °C for 12–16 h before the analysis. The ABTS radical solution was properly diluted with ethanol 70% to obtain an absorbance (λ = 734 nm) of 0.7 ± 0.02. The freeze-dried protein hydrolysates were dispersed in ultrapure water at concentrations ranging from 0.78 to 12.5 mg mL^−1^. The reduction of radical ABTS was monitored at the start and after 50 min from the beginning of the reaction. Two technical replicates for each sample were performed. The antioxidant power of samples was expressed as a percentage of inhibition, and an IC_50_ value was calculated from the regression curve plotting different concentrations of hydrolysates against the percentage of activity, and expressed as the mean ± SD. Data were analyzed as described in Section 2.4

### 2.6. Antibacterial Assays

The antibacterial activity of scotta hydrolysates was tested against six bacterial strains (see Table 1) belonging to three different species, namely, *Listeria monocytogenes* (four strains), *Staphylococcus aureus* and *Salmonella bongori*. The strains, stored at −80 °C, were thawed and precultured in Brain Heart infusion broth medium (BHI, WVR, Milano, Italy) for 24 h at 37 °C. Overnight cultures were then used to prepare microbial inoculation used for the test. One milliliter of overnight culture was centrifuged at 14,000× *g* for 2 min, then the pellets were resuspended in saline solution until reaching 0.2 optical density (OD) (~8 log10 CFU mL^−1^).

The lyophilized hydrolysates (BSPH, PSPH) and non-hydrolysate (CTRL) were weighed and dissolved in Brain Heart Infusion broth (BHI), giving a final concentration of 100 mg mL^−1^. The solutions were then filter-sterilized on a 0.22 µm filter (Sartorius). Aliquots of 100 µL of filtered BSPH, PSPH, CTRL, and BHI without hydrolysates (BHI-WH) as positive control were dispensed on 96-wells microtiter plates and inoculated with 5 µL of the bacterial suspension as previous prepared. Four wells for each strain and for each solution (BSPH, PSPH, CTRL and BHI-WH) were set up. The antibacterial assay was performed separately on separate microtiter plates for each sample and for each batch.

As blank samples, 100 µL BHI-hydrolysate and BHI-WH solutions before incubation were used. The microtiter plates were then incubated at 37 °C for 24 h and growth was measured automatically every 30 min at OD600 using a SPECTROstar nano microplate spectrophotometer reader (BMG Labtech, Ortenberg, Germany). Each growth curve was fitted by the primary model of Baranyi and Roberts [39] wrapped in DMFit Excel add-in [40], that was utilized also to evaluate the maximum specific growth rate (μ), the duration of lag phase (λ) according to Petretto et al. [38]. The 1000XOD absorbance values were log transformed to calculate the growth parameters with DMFIT add-in. Analysis of variance (ANOVA) was performed separately for each bacterial strain tested, using as factor the four treatments: BSPH, PSPH, CTRL, and BHI-WH to evaluate the influence of the two hydrolysates on the values of maximum specific growth rate (μmax) and lag phase (λ). When a significant effect was observed (*p* < 0.05), the differences between means were separated using the Tukey-Kramer multiple comparisons test. SPSS software, version 22, was used to conduct the statistical analyses.

### 2.7. Gel Permeation Chromatography

The freeze-dried scotta hydrolysates and control samples were reconstituted in water at 6 mg mL^−1^ and filtered on a 0.2 µm filter, then analyzed by Gel Permeation high performance liquid Chromatography (GPC) using an Agilent 1260 HPLC system equipped with a DAD detector. The separation was performed at 25 °C in isocratic mode with a mobile phase composed of 70:30 ACN:H_2_O with 0.1% TFA, flowing continuously at 0.5 mL min^−1^ through a Phenomenex Yarra SEC-2000 column (300 × 7.8 mm; pore size 3 µm). Samples were filtered through 0.2 µm nylon filter and 20 µL were injected. The analytical signal was acquired for 30 min at 214 nm. A calibration of molecular weights (MW) was obtained by acquiring the retention volume of the following pure standards covering a MW range from 181.19 to 66,500 Da: Tyr, Asp-Glu, Leu-Trp-Met-Arg, bacitracin, aprotinin, α-La, β-Lg, BSA. The obtained linear model was adopted to determine the MW distribution of the hydrolysates. The results were expressed as relative abundance by summing the areas of the peaks detected at different molecular weight (obtained by the calibration curve) ranges (1 kDa, 1–5 kDa, 5–10 kDa and >10 kDa), as previously reported by [41]. Data were analyzed as described in Section 2.4.

A semi-preparative GPC step was further performed on the above-described samples by injecting 100 µL in the same conditions above described. The fraction corresponding to a calculated MW between 5000 and 330 Da was collected for further LC-MS/MS analysis.

### 2.8. LC-MS/MS Analysis

Fractions obtained from nine different semi-preparative GPC runs (three of CTRL, three of BSPH and three of PSPH hydrolysates) were brought to dryness and reconstituted in 0.2% formic acid. The peptide mixture concentration was estimated by measuring absorbance at 280 nm with a NanoDrop 2000 spectrophotometer (Thermo Scientific, San Jose, CA, USA), using dilutions of the MassPREP *E. coli* Digest Standard (Waters, Milford, MA, USA) to generate a calibration curve. Peptide concentration was adjusted to 1 µg µL^−1^. Two technical replicates for each sample were performed.

LC-MS/MS analyses were carried out using a Q Exactive mass spectrometer (Thermo Scientific) interfaced with an UltiMate 3000 RSLCnano LC system (Thermo Scientific). After loading, peptide mixtures (4 μg per run) were concentrated and desalted on a trapping precolumn (Acclaim PepMap C18, 75 μm × 2 cm nanoViper, 3 μm, 100 Å, Thermo Scientific), using 0.2% formic acid at a flow rate of 5 μL min^−1^. The peptide separation was performed at 35 °C using a C18 column (EASY-Spray column, 50 cm × 75 μm ID, PepMap C18, 3 μm, Thermo Scientific), using a linear gradient of 245 min from 5% to 37.5% of eluent B (0.1% formic acid in 80% acetonitrile) in eluent A (0.1% formic acid), at a flow rate of 250 nL min^−1^. MS data were acquired using a data-dependent Top12 method dynamically choosing the most abundant precursor ions from the survey scan, under direct control of the Xcalibur software (version 1.0.2.65 SP2, Thermo Fisher Scientific), where a full-scan spectrum (from 300 to 1700 *m*/*z*) was followed by tandem mass spectra (MS/MS). The instrument was operated in positive mode with a spray voltage of 1.8 kV and a capillary temperature of 275 °C. Survey and MS/MS scans were performed in the Orbitrap with resolution of 70,000 and 17,500 at 200 *m*/*z*, respectively. The automatic gain control was set to 1,000,000 ions and the lock mass option was enabled on a protonated polydimethylcyclosiloxane background ion as an internal recalibration for accurate mass measurements. The dynamic exclusion was set to 30 s. Higher Energy Collisional Dissociation (HCD), performed at the far side of the C-trap, was used as fragmentation method by applying a 25 eV value for normalized collision energy. An isolation width of *m*/*z* 2.0. Nitrogen was used as the collision gas.

Peptide identification was performed using Proteome Discoverer (version 1.4; Thermo Scientific), with Sequest-HT as the search engine for protein identification, according to the following criteria: Database: custom, obtained by merging *Bos Taurus* and *Ovis aries* downloaded from UniprotKB, (release 2019_01); Precursor mass tolerance: 10 ppm; Fragment mass tolerance: 0.02 Da; Dynamic modification: methionine oxidation, Asparagine/Glutamine, Arginine deamidation, Serine/Threonine/Tyrosine phosphorylation), and Percolator for peptide validation (FDR < 1% based on peptide q-value). Results were filtered in order to keep only Rank 1 peptides, and protein grouping was allowed according to the maximum parsimony principle. Protein abundance was expressed by means of the normalized spectral abundance factor (NSAF). NSAF was calculated to evaluate the relative abundance of each protein and peptide, and “log ratio (log R)” values (log_2_ NSAF group a/NSAF group b) were obtained to estimate the fold changes of peptides between experimental groups expressed as base 2 on a logarithmic scale [42,43]. In this approach, the spectral counts of each peptide were divided by its length and normalized to the average number of spectral counts in a given analysis. In order to eliminate discontinuity due to SpC = 0, a correction factor, set to 0.01, was used.

Peptides showing log ratio >1.5 or <−1.5 were considered as differentially abundant between groups. A two-tailed *t*-test was applied, using in house software to evaluate the statistical significance of differences between groups. The differentially abundant peptides were then evaluated using the “profiles of potential biological activity” analysis, available on BIOPEP [44] to find within them any sequence with known DPP-IV inhibitory, antioxidant and antibacterial activity.

## 3. Results and Discussion

### 3.1. DPP-IV Inhibitory, Antioxidant Activity and GPC Profile of the Selected Hydrolysates

The DPP-IV inhibitory and ABTS activity of the obtained hydrolysates and of the control samples are showed in Table 2. The P32/98 positive control showed a IC_50_ of 1.395 ± 0.007 × 10^−3^ mg mL^−1^. In contrast, the retentate control samples (CTRL) did not show a measurable inhibition. BSPH had a significantly higher DPP-IV inhibitory activity compared to PSPH (*p* = 0.0381). The obtained DPP-IV inhibitory activity was lower than that previously described for milk and whey hydrolysates obtained from WPC, WPI of different species [45,46,47,48,49,50]. However, the observed activity was just about one order of magnitude less than that measured on hydrolysates obtained from pure β-lactoglobulin [41]. In addition, a DPP-IV inhibitory activity on hydrolysates from scotta has never been measured before, leading to the consideration of this matrix as a candidate substrate for the industrial production of DPP-IV inhibitory peptides.

Further, the obtained data confirmed that enzymatic hydrolysis is a suitable way to increase the radical scavenging ability of sheep milk by-products [51]. In fact, BSPH showed a higher antioxidant activity compared to the control. Despite that, the hydrolysates did not differ regarding this property. A similar pattern was found by Monari et al. [15] for bovine scotta hydrolysates, which showed that the antioxidant activity of hydrolysates obtained using bromelain and pancreatin enzymes, did not differ significantly, even using different E:S ratios, both in unconcentrated bovine scotta and retentates.

**Figure 1:**
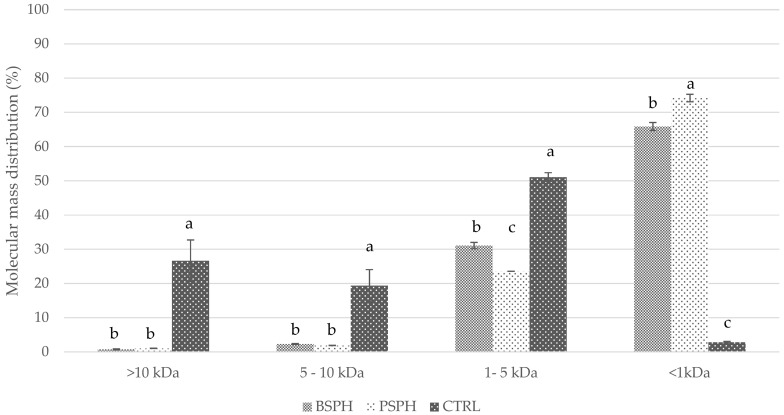
Distribution of the relative abundance (%) according to molecular weight obtained by gel permeation chromatography (GPC), and the comparison among BSPH, PSPH and CTRL. Values (*n* = 3) with the same letter do not differ significantly from each other according to LSD test (*p* < 0.05).

Figure 1 shows the peptide distribution in terms of relative abundance according to the molecular weight obtained by gel permeation chromatography (GPC), and the comparison among BSPH, PSPH and CTRL.

As expected, most of BSPH and PSPH components were low molecular weight peptides (<1 kDa), whilst the high and medium molecular weight peptides (>10 kDa, 5–10 kDa, and 1–5 kDa) were more abundant in the control samples, which conversely showed a very low contribution of components with MW < 1 kDa (2.82 ± 0.21%). The amount of the 1–5 kDa fraction, even lower than the control, was significantly higher in BSPH (31 ± 0.9%) than in PSPH (23.56 ± 0.02%). Conversely, pancreatin in our system was more effective in producing peptides with low MW (74 ± 1%), compared to bromelain (66 ± 1%). Since pancreatin contains a mixture of proteases including chymotrypsin, trypsin and elastase [52], we suppose that in our system it exerted a more generalized proteolytic behavior than bromelain, which conversely has been reported to be less effective in producing free amino acids [15]. The specificity of bromelain may be responsible of the higher DPP-IV inhibitory activity measured in our hydrolysates.

### 3.2. Antibacterial Activities of Hydrolysates

**Figure 2:**
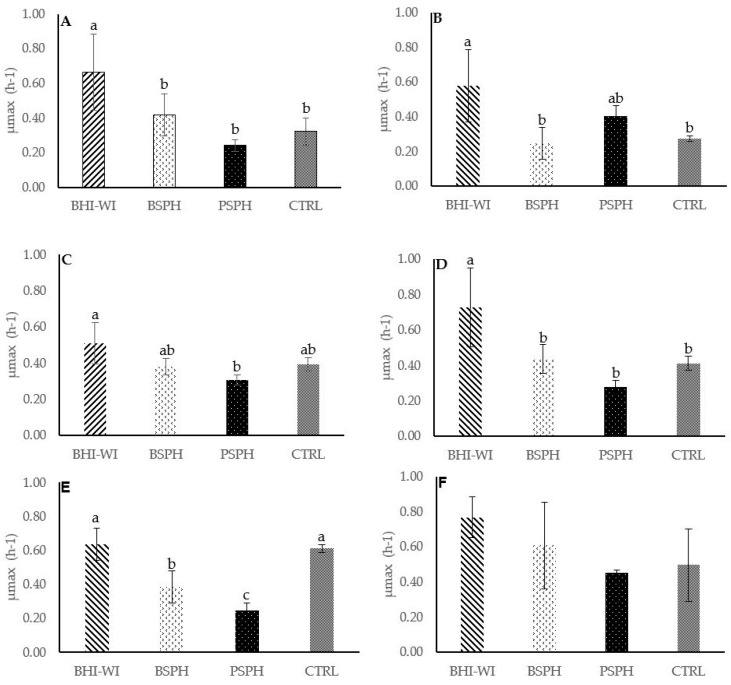
Effect of the different scotta-hydrolysates at concentration of 100 mg mL^−1^ on the maximum growth rate (µmax) of bacteria strains target. BHI-WH, Brain Heart infusion broth medium without hydrolysates; BSPH, Bromelain filter sterilized hydrolysate; PSPH, Pancreatin filter sterilized hydrolysate; CTRL, Scotta not hydrolysate filter sterilized). (Panel (**A**–**F**): *Listeria monocytogenes* B (**A**); *L. monocytogenes* C (**B**); *L. monocytogenes* 20,600 DSMZ (**C**); *L. monocytogenes* E (**D**); *Staphylococcus aureus* 20,231 DSMZ (**E**), *Salmonella bongori* 13,772 DSMZ (**F**)). Different lowercase letters above the bar indicate statistically significant differences between different treatments (*p <* 0.001).

The enzymatic hydrolysates obtained from ovine scotta tested did not show a complete inhibitory or bactericidal effect on the target microorganisms at the concentration tested (100 mg mL^−1^). However, the hydrolysates showed a variable but slightly inhibitory effect depending on the species or strain of bacteria tested. In particular, PSPH decreased significantly (*p* < 0.001), the maximum specific growth rate (μmax) respect to control being half the values in all bacteria tested except for *Salmonella bongori* 13,772 DSMZ strain, where the difference of its growth rate respect to control was not significant (see Figure 2A–F). The influence of BSPH was strain-dependent, decreasing significantly the μmax of *Listeria monocytogenes* B, *Listeria monocytogenes* D and *Staphylococcus aureus* 20,231 DSMZ, whereas the BSPH did not influence the μmax of *L. monocytogenes* 20,600 DSMZ, *L. monocytogenes* B and *Salmonella bongori* 13,772 DSMZ. An intriguing result was obtained with the CTRL, that decreased the μmax of *Listeria monocytogenes* B, *L. monocytogenes* C and *L. monocytogenes* E. All three strains were isolated from ovine ricotta cheese. Regarding the effect of scotta hydrolysates tested, no effect was observed on the lag time (λ) of *L. monocytogenes* C, *L. monocytogenes* E, *S. aureus* 20,231 DSMZ and *S. bongori* 13,772 DSMZ. An opposite effect of PSPH on lag time with respect to μmax was observed on *L. monocytogenes* 20,600 DSMZ (λ: 3.96 h) and *L. monocytogenes* B (λ: 1.42 h) strains, with a lag time for each bacterial strains that did not differ significantly from the control (3.83 h and 3.39 h for *L. monocytogenes* 20,600 DSMZ and *L. monocytogenes* B control respectively), but was significantly different from the other two treatments (BSPH and CTRL). Overall, all treatments reduced the bacterial density, confirming that scotta hydrolysates negatively influenced the growth of the bacteria tested. Contrasting with our results, Lestari et al. [36] revealed a strong antimicrobial activity of goat milk protein hydrolysate by using bromelain as a hydrolyzing agent. Indeed, the minimum inhibitory concentrations of these hydrolysates against *S. aureus* and *Escherichia coli* were below 100 ppm. Bovine β-LG and α-LA were previously treated with pancreatin, and the resulting hydrolysates were shown to possess antimicrobial activity [53].

### 3.3. LC-MS/MS Analysis of Scotta Hydrolysates

The LC-MS/MS analysis of the GPC fraction of the hydrolysates (BSPH, PSPH and CTRL) described in Section 2.8 allowed acquisition of 12,000 spectra from each run. A total of 58 ± 7 proteins and 547 ± 24 peptides were identified in BSPH, while 75 ± 6 proteins and 559 ± 33 peptides were identified in PSPH and further 83 ± 15 proteins and 1506 ± 381 peptides were identified in the CTRL samples (see Supplementary Materials, Sheet S1).

Considering BSPH vs. CTRL, a total of 29 proteins showed significant differences (*p* ≤ 0.05). Twenty-one of them were more abundant in BSPH, while eight were more abundant in CTRL (see Supplementary Materials, Sheet S2). The differential analysis of BSPH vs. CTRL highlighted 751 significant peptides (*p* ≤ 0.05). Of these, 388 were more abundant in BSPH samples, whilst 363 were more abundant in CTRL samples (see Supplementary Materials, Sheet S3).

Considering the available literature, differential peptides in BSPH were investigated to find sequences with reported DPP-IV inhibition, and antioxidant and antibacterial activity. The candidate peptides were further evaluated using the tool “profiles of potential biological activity” analysis, available on BIOPEP [44].

This approach highlighted 97 differential peptides containing at least one of the following sequences with known biological activity: LPQNI, VLGP, VLVLDTDYK, IPAVF, IPA, LKPTPEG, YPVEPF, YQEPVLGPVR, YVEEL, LDTDYKK, IDALNENK, KVAGT, AASDISLLDAGSAPLR, and ALK (see Table 3). All these peptides were attributable to β-lactoglobulin protein (P67976), except for LPQNI, VLGP, YPVEPF, YQEPVLGPVR derived from β-casein protein (P11839), and the tripeptide IPA originating from k-casein (P02669). In the following text and in the tables the active sequences contained in the identified peptides will be highlighted with bold characters.

The differential peptides within the DPP-IV sequence showed a length ranging from eight to twenty-eight amino acid residues. The shortest peptide was **LDTDYKK**Y from β-lactoglobulin with an estimated MW of 1044.52 Da (Log R = 2.38), whilst the longest was AIPPKKDQDKTE**IPA**INTIASAEPTVHS released from k-casein with an estimated MW of 3051.55 Da (Log R = 2.78).

Considering PSPH vs. CTRL, a total of 32 proteins showed a significant difference (*p* ≤ 0.05). Twenty-five of them were more abundant in PSPH samples, while seven were more abundant in CTRL samples (see Supplementary Materials, Sheet S4). The peptide differential analysis of PSPH vs. CTRL highlighted 667 significant peptides (*p* ≤ 0.05). Of these, 294 were differential in PSPH, and 373 were more abundant in the CTRL profile (see Supplementary Materials, Sheet S5).

The “profiles of potential biological activity” analysis on BIOPEP revealed 75 differential peptides containing at least one of the sequences previously observed in BSPH (see Table 4). The sequences with DPP-IV inhibitory activity were encrypted in peptides of 9 to 22 amino acid residues. In detail, K**IDALNENK** and **ALK**ALPMHI appeared the shortest peptides originating from β-lactoglobulin with an estimated MW of 1044.56 Da (Log R = 2.84) and MW of 992.59 Da (Log R = 2.03) respectively. Furthermore, the longer peptide identified was KDQDKTE**IPA**INTIASAEPTVH deriving from k-casein, with an estimated MW of 2884.55 Da (Log R =5.07).

**Figure 3:**
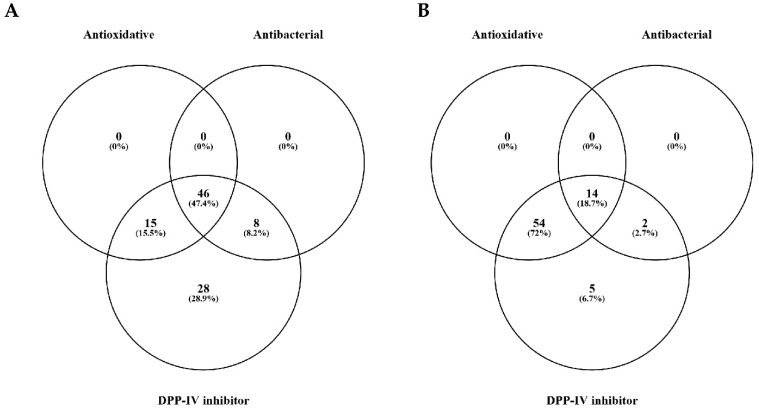
Distribution of the differential peptides more abundant in BSPH vs. CRTL (**A**) and PSPH vs. CRTL (**B**), according to their putative biological activities (DPP-IV inhibition, antioxidative and antibacterial properties).

Venn diagrams were used to evaluate the number of differential peptides, more abundant in BSPH vs. CRTL and PSPH vs. CRTL (Figure 3A,B, respectively), with potential biological activities.

Venn diagrams highlighted that none of the peptides contained sequences with only antibacterial or antioxidant known activity (Figure 3A,B). Interestingly, the 72% of the peptides in PSPH compared to the CTRL, contained sequences associated with antioxidant and DPP-IV inhibitory activity (Figure 3B). Moreover, a 5.6-fold higher number of peptides containing sequences with only DPP-IV inhibitory activity was found in BSPH vs. CTRL compared to PSPH vs. CTRL.

Furthermore, the differential analysis of BSPH vs. PSPH showed 82 proteins in total and 29 differentials (*p* ≤ 0.05). Among them, 21 were more abundant in BSPH, while eight were more abundant in PSPH (see Supplementary Materials, Sheet S6). The peptide analysis indicated 1181 peptides, and 752 were significantly differential (*p* ≤ 0.05). Among them, 388 peptides were more abundant in BSPH, while 364 were more abundant in PSPH (see Supplementary Materials, Sheet S7).

A total of 208 differential peptides contained sequences with known DPP-IV inhibitory activity (see Table 5).

**Figure 4:**
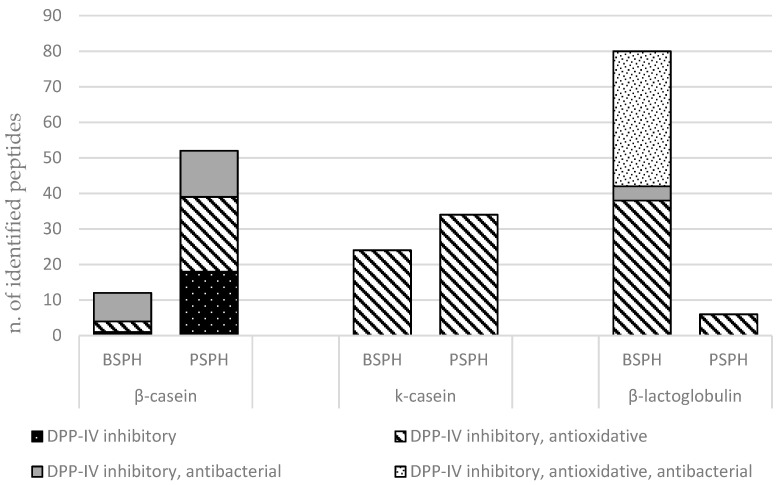
Number of differential peptides (BSPH vs. PSPH) grouped by the proteins and biological activities.

Figure 4 groups differential peptides (BSPH vs. PSPH) generated by the same protein and dividing the components according to reported biological activity. Histograms show, for each protein (β-casein, k-casein, and β-lactoglobulin), the peptides more abundant in BSPH or in PSPH, respectively.

Sixty-four differential peptides were derived from β-casein, 52 of which were more abundant in PSPH, and 12 were more abundant in BSPH (Figure 4). Fifty-eight differential peptides were originated from k-casein 24, of which were more abundant in BSPH and 34 were more abundant in PSPH.

Interestingly, the number of differential peptides derived from β-lactoglobulin was differently distributed between BSPH and PSPH. In fact, a total of 86 differential peptides derived from β-lactoglobulin were identified, 80 of them more abundant in BSPH and six in PSPH. This result could help to interpret the higher DPP-IV inhibitory activity showed in vitro by BSPH compared to PSPH. Moreover, none of the identified peptides derived from α-lactalbumin. Power et al. [50], found in silico a three-fold higher content of peptide sequences with potential DPP-IV inhibitory activity in β-lactoglobulin compared to α-lactalbumin. Furthermore, Tulipano et al. [54] found by in silico analysis that bovine β-lactoglobulin was a better source of DPP-IV inhibitory peptides compared with α-lactalbumin after treatment with digestive proteases.

**Table 3:** Analysis of differential peptides of BSPH vs. CTRL (Log R ≥ 1.5 and Log R ≤ −1.5).

ID Protein	Identified Peptide	Log_2_ BSPH vs. CTRL	Activity	Reference
β-casein	GPIPNS**LPQNI**LPLT^79–94^ GPIPNS**LPQNI**LPLTQ^79–95^	3.82 3.78	Antioxidative; DPP-IV inhibitory;	[55,56]
β-casein	YQEP**VLGP**VR^206–215^	2.1	Antioxidative; DPP-IV inhibitory;	[57,58,59,60,61,62,63,64,65]
β-lactoglobulin	**VLVLDTDYK**^110–118^ **VLVLDTDYK**K^110–119^ **VLVLDTDYK**KY^110–120^ **VLVLDTDYK**KYL^110–121^ **VLVLDTDYK**KYLL^110–122^ K**VLVLDTDYK**KY^109–120^ ENK**VLVLDTDYK**K^107–119^ ENK**VLVLDTDYK**KY^107–120^ ENK**VLVLDTDYK**KYL^107–121^ DALENK**VLVLDTDYK**K^104–119^ DALENK**VLVLDTDYK**KY^104–120^ IDALENK**VLVLDTDYK**K^103–119^ IDALENK**VLVLDTDYK**KY^103–120^	2.21 3.05 3.42 2.72 2.17 1.67 2.48 3.45 2.18 1.90 2.43 2.33 3.05	Antioxidative; Antibacterial; DPP-IV inhibitory;	[66,67,68,69,70,71,72,73]
β-lactoglobulin	**IPAVF**KIDALNENK^96–109^ TK**IPAVF**KIDALNENK^94–109^	2.43 1.83	Antioxidative; Antibacterial; DPP-IV inhibitory;	[26,67,68,71,74,75,76]
k-casein	DQDKTE**IPA**INTIASAEPTVHS^134–155^ AIPPKKDQDKTE**IPA**INT^128–145^ E**IPA**INTIASAEPTVHS^139–155^ IPPKKDQDKTE**IPA**INTIA^129–147^ **IPA**INTIASAEPTVHS^140–155^ IPPKKDQDKTE**IPA**IN^129–144^ IPPKKDQDKTE**IPA**INT^129–145^ PPKKDQDKTE**IPA**INTIAS^130–148^ AIPPKKDQDKTE**IPA**INTIA^128–147^ AIPPKKDQDKTE**IPA**IN^128–144^ KDQDKTE**IPA**INT^132–145^ KDQDKTE**IPA**INTIA^132–147^ E**IPA**INTIASAEPTVH^139–154^ DQDKTE**IPA**INTIAS^134–148^ **IPA**INTIASAEPTVH^140–154^ DQDKTE**IPA**INTIASAEPTVH^144–154^ TE**IPA**INTIASAEPTVHS^138–155^ PPKKDQDKTE**IPA**InTIASAEP^130–151^ AIPPKKDQDKTE**IPA**INTIASAEPTVHS^128–155^ DQDKTE**IPA**INTI^134–146^ KDQDKTE**IPA**INTIASAEPTVHS^133–155^ PPKKDQDKTE**IPA**INTIA^130–147^ KDQDKTE**IPA**IN^133–144^ DQDKTE**IPA**INTIA^134–147^	5.40 5.39 5.28 4.69 4.62 4.49 4.46 3.96 3.90 3.84 3.59 3.46 3.37 3.29 3.22 3.14 2.99 2.87 2.78 2.59 2.33 2.31 1.84 1.50	DPP-IV inhibitory;	[26,41,75,77,78]
β-lactoglobulin	VYVEE**LKPTPEG**^59–70^ E**LKPTPEG**NLEILLQ^63–77^ EE**LKPTPEG**NL^62–72^ VYVEE**LKPTPEG**NL^59–72^ VYVEE**LKPTPEG**NLE^59–73^ EE**LKPTPEG**NLEILL^62–76^	2.10 2.03 1.99 1.74 1.69 1.67	Antioxidative; DPP-IV inhibitory;	[31]
β-casein	EMPFPK**YPVEPF**T^123–135^ MPFPK**YPVEPF**TE^124–136^ EMPFPK**YPVEPF**TE^123–136^ MPFPK**YPVEPF**T^124–135^ MPFPK**YPVEPF**TES^124–137^ EMPFPK**YPVEPF**TES^123–137^ MPFPK**YPVEPF**^124–134^ EMPFPK**YPVEPF**^123–134^	4.23 4.01 3.96 3.90 3.39 3.15 1.93 1.90	Antibacterial; DPP-IV inhibitory;	[27,65,79,80,81]
β-casein	**YQEPVLGPVR** ^208–212^	2.10	DPP-IV inhibitory; Antioxidative;	[58,60,62,64]
β-lactoglobulin	**VYVEEL**KPTPEG^59–70^ **VYVEEL**KPTPEGNL^59–72^ **VYVEEL**KPTPEGNLE^59–73^	2.10 1.74 1.69	DPP-IV inhibitory; Antioxidative; Antibacterial	[24,82,83]
β-lactoglobulin	ENKVLV**LDTDYKK**Y^107–120^ VLV**LDTDYKK**Y^111–120^ **LDTDYKK**YL^113–121^ VLV**LDTDYKK**^112–120^ IDALNENKVLV**LDTDYKK**Y^102–120^ LV**LDTDYKK**Y^111–120^ VLV**LDTDYKK**YL^112–121^ V**LDTDYKK**Y^112–120^ LV**LDTDYKK**YL^111–121^ **LDTDYKK**YLL^113–122^ ENKVLV**LDTDYKK**^107–119^ DALNENKVLV**LDTDYKK**Y^103–120^ **LDTDYKK**Y^113–120^ IDALNENKVLV**LDTDYKK**^102–119^ LV**LDTDYKK**^111–119^ ENKVLV**LDTDYKK**YL^107–121^ VLV**LDTDYKK**YLL^112–122^ DALNENKVLV**LDTDYKK**^103–119^ KVLV**LDTDYKK**Y^109–120^	3.45 3.42 3.18 3.05 3.05 2.94 2.72 2.62 2.52 2.48 2.48 2.43 2.38 2.33 2.23 2.18 2.17 1.90 1.67	Antioxidative; Antibacterial; DPP-IV inhibitory;	[44,84]
β-lactoglobulin	IPAVFK**IDALNENK**^96–109^ **IDALNENK**VLVLDTDYKKY^102–120^ **IDALNENK**VL^102–111^ TKIPAVFK**IDALNENK**^94–109^ **IDALNENK**VLVLDTDYKK^102–119^ KIPAVFK**IDALNENK**^95–109^ K**IDALNENK**^101–109^ VFK**IDALNENK**^99–109^ **IDALNENK**V^102–110^	3.09 3.05 2.60 2.46 2.33 2.30 2.21 1.99 1.96	DPP-IV inhibitory; Antioxidative; Antibacterial;	[44,85]
β-lactoglobulin	LDIQ**KVAGT**WHS^27–39^	1.92	DPP-IV inhibitory; Antioxidative;	[86]
β-lactoglobulin	**AASDISLLDAQSAPLR**^43–59^ LAMA**ASDISLLDAQSAPLR**^39–59^ M**AASDISLLDAQSAPLR**^42–59^ **AASDISLLDAQSAPLR**V^43–59^	2.57 2.37 1.88 1.53	DPP-IV inhibitory;	[68,71]
β-lactoglobulin	TPEVDNEALEKFDK**ALK**^143–159^ TPEVDNEALEKFDK**ALK**A^142–160^ DNEALEKFDK**ALK**^147–159^ EVDNEALEKFDK**ALK**^145–159^	4.03 2.92 2.38 2.01	DPP-IV inhibitory; Antioxidative;	[76]

The active sequences contained in longer peptides are highlighted with bold characters.

**Table 4:** Analysis of differential peptides of PSPH vs. CTRL (Log R ≥ 1.5 and Log R ≤ –1.5).

ID Protein	Identified Peptide	Log_2_ PSPH vs. CTRL	Activity	Reference
β-casein	TGPIPNS**LPQNI**LPL^78–92^	2.53	DPP-IV inhibitory; Antioxidative;	[55,56]
β-casein	QEP**VLGP**VRGPFPI^207–220^ QEP**VLGP**VRGPFP^207–219^ YQEP**VLGP**VRGPFPI^206–220^ LYQEP**VLGP**VRGPFPI^205–220^ EP**VLGP**VRGPFPI^208–220^	4.06 3.44 2.45 1.91 1.85	DPP-IV inhibitory;	[57,58,59,60,61,62,63,64,65]
β-lactoglobulin	KIDALNENK**VLVLDTDYK**^101–118^ KIDALNENK**VLVLDTDYK**K^101–119^ **VLVLDTDYK**KY^112–120^ IDALNENK**VLVLDTDYK**K^102–119^ **VLVLDTDYK**KYL^112–121^ KIDALNENK**VLVLDTDYK**KY^101–120^	2.83 2.50 2.20 2.04 1.93 1.57	DPP-IV inhibitory; Antioxidative; Antibacterial;	[66,67,68,69,70,71,72,73]
β-lactoglobulin	**IPAVF**KIDALNENK^96–109^	1.76	DPP-IV inhibitory; Antioxidative; Antibacterial;	[26,67,68,71,74,75,76]
k-casein	KDQDKTE**IPA**INTIASAEPT^133–152^ KDQDKTE**IPA**INT^133–144^ TE**IPA**INTIASAEPTVH^138–154^ KDQDKTE**IPA**INTIA^133–146^ KDQDKTE**IPA**INTIASAEPTVH^133–154^ DQDKTE**IPA**INTIASAEPT^134–152^ DQDKTE**IPA**INTIASAEPTVH^134–154^ KDQDKTE**IPA**IN^133–143^ KDQDKTE**IPA**INTIAS^133–147^ KDQDKTE**IPA**I^133–142^ E**IPA**INTIASAEPTVH^139–154^ KDQDKTE**IPA**INTI^133–145^ DQDKTE**IPA**INTIAS^134–147^ DQDKTE**IPA**INTIA^134–146^ DQDKTE**IPA**INTI^134–145^ MAIPPKKDQDKTE**IPA**^127–142^ AIPPKKDQDKTE**IPA**IN^128–144^ AIPPKKDQDKTE**IPA**INTIA^128–147^ PPKKDQDKTE**IPA**IN^130–144^ MAIPPKKDQDKTE**IPA**INTIA^127–147^ AIPPKKDQDKTE**IPA**^128–142^	5.99 5.85 5.17 5.13 5.07 5.05 5.04 4.39 3.96 3.92 3.91 3.90 3.65 3.42 3.32 2.95 2.70 2.64 2.32 2.09 1.68	DPP-IV inhibitory; Antioxidative;	[26,41,75,77,78]
β-lactoglobulin	VEE**LKPTPEG**NLE^61–73^ VEE**LKPTPEG**NLEI^61–74^ VEE**LKPTPEG**NLEILLQK^61–78^ VEE**LKPTPEG**NLEIL^61–76^ YVEE**LKPTPEG**NLE^60–73^ VEE**LKPTPEG**DLE VYVEE**LKPTPEG**N^59–71^ VYVEE**LKPTPEG**NLE^59–73^ YVEE**LKPTPEG**N^60–70^ YVEE**LKPTPEG**NLEI^59–74^ YVEE**LKPTPEG**NLEILLQK^59–78^ YVEE**LKPTPEG**NLEIL^59–75^ VYVEE**LKPTPEG**NLEILLQK^58–78^ VEE**LKPTPEG**NL^60–72^ RVYVEE**LKPTPEG**NLEILLQK^58–78^ VYVEE**LKPTPEG**NL^58–72^	3.51 3.35 2.97 2.96 2.89 2.64 2.64 2.63 2.61 2.45 2.28 2.27 2.02 1.93 1.87 1.76	DPP-IV inhibitory; Antioxidative;	[31]
β-casein	EMPFPK**YPVEPF**^129–134^	1.93	DPP-IV inhibitory; Antibacterial;	[27,65,79,80,81]
β-casein	**YQEPVLGPVR**GPFPI^208–217^ L**YQEPVLGPVR**GPFPI^206–215^	2.45 1.91	DPP-IV inhibitory; Antioxidative;	[58,60,62,64]
β-lactoglobulin	**VYVEEL**KPTPEGN^59–71^ **VYVEEL**KPTPEGNLE^59–73^ **VYVEEL**KPTPEGNLEILLQK^59–78^ R**VYVEEL**KPTPEGNLEILLQK^58–78^ **VYVEEL**KPTPEGNL^59–72^	2.64 2.63 2.02 1.87 1.76	DPP-IV inhibitory; Antioxidative;	[24,82,83]
β-lactoglobulin	KIDALNENKVLV**LDTDYKK**^101–119^ VLV**LDTDYKK**Y^112–120^ V**LDTDYKK**YL^112–121^ IDALNENKVLV**LDTDYKK**^102–119^ VLV**LDTDYKK**YL^112–121^ KIDALNENKVLV**LDTDYKK**Y^101–120^	2.50 2.20 2.12 2.04 1.93 1.57	DPP-IV inhibitory; Antibacterial; Antioxidative;	[44,84]
β-lactoglobulin	K**IDALNENK**V^101–110^ K**IDALNENK**^101–109^ K**IDALNENK**VLVLDTDYK^101–118^ K**IDALNENK**VLVLDTDYKK^101–119^ **IDALNENK**VLVLDTDYKK^102–119^ IPAVFK**IDALNENK**^96–109^ K**IDALNENK**VLVLDTDYKKY^100–120^	3.39 2.84 2.83 2.50 2.04 1.76 1.57	DPP-IV inhibitory; Antioxidative;	[44,85]
β-lactoglobulin	GLDIQ**KVAGT**WH^27–38^	1.73	DPP-IV inhibitory; Antioxidative;	[86]
β-lactoglobulin	SLAM**AASDISLLDAQSAPLR**V^39–59^ SLAM**AASDISLLDAQSAPLR**^39–58^	2.56 2.21	DPP-IV inhibitory; Antibacterial;	[68,71]
β-lactoglobulin	**ALK**ALPMHI^157–165^	2.03	DPP-IV inhibitory; Antioxidative;	[76]

The active sequences contained in longer peptides are highlighted with bold characters.

**Table 5:** Analysis of differential peptides of BSPH vs. PSPH (Log R ≥ 1.5 and Log R ≤ −1.5).

ID Protein	Identified Peptide	Log_2_ BSPH vs. PSPH	Activity
β-casein	GPIPNS**LPQNI**LPLT^79–93^ GPIPNS**LPQNI**LPLTQ^79–94^ LVYPFTGPIPNS**LPQNI**LPLTQTPVVVPPFLQPEIMGVPK^73–112^ S**LPQNI**LPLTQTPVVVPPFLQPEIMGVPKVKET^72–116^ TGPIPNS**LPQNI**LPLTQTPVVVPPFLQPEIMGVPKVKETMVPKH^78–121^ S**LPQNI**LPLTQTPVVVPPFLQPEIMGVPKVKETMVPKH^72–121^ S**LPQNI**LPLTQTPVVVPPFLQPEIMGVPKVK^72–114^ S**LPQNI**LPLTQTPVVVPPFLQPEIMGVPK^72–120^ FTGPIPNS**LPQNI**LPLTQTPVVVPPFLQPEIMGVPKVKETMVPKH^77–121^ FTGPIPNS**LPQNI**LPLTQTPVVVPPFLQPEIMGVPKVKETMVPK^77–120^ S**LPQNI**LPLTQTPVVVPPFLQPEIMGVPKVKETMVPK^72–120^ TGPIPNS**LPQNI**LPLTQTPVVVPPFLQPEIMGVPKVKETMVPK^78–120^	3.82 3.78 −1.54 −1.62 −1.69 −1.91 −1.92 −1.98 −2.44 −3.44 −3.47 −3.53	DPP-IV inhibitory; Antioxidative;
β-casein	YQEP**VLGP**VR^206–215^ YQEP**VLGP**VRGPFP^206–219^ VLPVPQKAVPQRDMPIQAFLLYQEP**VLGP**VRGPFP^185–219^ LSLSQPKVLPVPQKAVPQRDMPIQAFLLYQEP**VLGP**V^178–214^ AVPQRDMPIQAFLLYQEP**VLGP**VRGPFPI^192–220^ SLSQPKVLPVPQKAVPQRDMPIQAFLLYQEP**VLGP**VRGPFPILV^179–222^ AVPQRDMPIQAFLLYQEP**VLGP**VRGPFP^192–219^ EP**VLGP**VRGPFPIIV^208–222^ EP**VLGP**VRGPFPILV^208–222^ EP**VLGP**VRGPFPI^208–220^ FLLYQEP**VLGP**VRGPFP^203–219^ VLPVPQKAVPQRDMPIQAFLLYQEP**VLGP**VRGPFPILV^185–222^ VLPVPQKAVPQRDMPIQAFLLYQEP**VLGP**VRGPFPI^185–220^ YQEP**VLGP**VRGPFPIIV^206–222^ YQEP**VLGP**VRGPFPILV^206–222^ VLPVPQKAVPQRDMPIQAFLLYQEP**VLGP**VRGPFPIL^185–221^ EP**VLGP**VRGPFPII^208–221^ EP**VLGP**VRGPFPIL^208–221^ EP**VLGP**VRGPFP^208–219^	2.10 −1.61 −1.64 −1.71 −1.75 −1.77 −1.80 −1.89 −1.89 −2.01 −2.10 −2.14 −2.24 −2.31 −2.31 −2.33 −2.59 −2.59 −2.98	DPP-IV inhibitory;
β-lactoglobulin	ENK**VLVLDTDYK**KY^107–118^ VL**VLDTDYK**KY^110–120^ **VLVLDTDYK**K^112–119^ IDALNENK**VLVLDTDYK**KY^102–120^ **VLVLDTDYK**KYL^112–120^ ENK**VLVLDTDYK**K^107–119^ DALNENK**VLVLDTDYK**KY^103–120^ IDALNENK**VLVLDTDYK**K^102–119^ **VLVLDTDYK**^112–118^ ENKVL**VLDTDYK**KYL^107–121^ **VLVLDTDYK**KYLL^112–120^ DALNENK**VLVLDTDYK**K^103–119^ K**VLVLDTDYK**KY^109–120^	3.45 3.42 3.05 3.05 2.72 2.48 2.43 2.33 2.21 2.18 2.17 1.90 1.67	DPP-IV inhibitory; Antioxidative; Antibacterial;
β-lactoglobulin	**IPAVF**KIDALNENK^106–109^ TK**IPAVF**KIDALNENK^104–109^ K**IPAVF**KIDALNENK^105–109^	3.09 2.46 2.30	DPP-IV inhibitory; Antioxidative; Antibacterial;
k-casein	DQDKTE**IPA**INTIASAEPTVHS^134–155^ AIPPKKDQDKTE**IPA**INT^128–145^ E**IPA**INTIASAEPTVHS^139–155^ IPPKKDQDKTE**IPA**INTIA^129–147^ **IPA**INTIASAEPTVHS^140–155^ IPPKKDQDKTE**IPA**IN^129–144^ IPPKKDQDKTE**IPA**INT^129–145^ PPKKDQDKTE**IPA**INTIAS^130–148^ AIPPKKDQDKTE**IPA**INTIA^128–147^ AIPPKKDQDKTE**IPA**IN^128–144^ KDQDKTE**IPA**INT^133–145^ KDQDKTE**IPA**INTIA^133–147^ E**IPA**INTIASAEPTVH^139–154^ DQDKTE**IPA**INTIAS^134–148^ **IPA**INTIASAEPTVH^140–154^ DQDKTE**IPA**INTIASAEPTVH^134–154^ TE**IPA**INTIASAEPTVHS^138–155^ PPKKDQDKTE**IPA**INTIASAEP^130–151^ AIPPKKDQDKTE**IPA**INTIASAEPTVHS^128–155^ DQDKTE**IPA**INTI^134–146^ KDQDKTE**IPA**INTIASAEPTVHS^133–155^ PPKKDQDKTE**IPA**INTIA^130–147^ KDQDKTE**IPA**IN^133–144^ DQDKTE**IPA**INTIA^134–147^ FMAIPPKKDQDKTE**IPA**INTIASAEPTVH^126–154^ MAIPPKKDQDKTE**IPA**INTIASAEPTVHSTPTTEAVVNAVDNP^127–169^ KTE**IPA**INTIASAEPTVH^137–154^ MAIPPKKDQDKTE**IPA**INTIASAEPTVHSTPTTEAVV^127–163^ IPPKKDQDKTE**IPA**INTIASAEPTVH^129–154^ MAIPPKKDQDKTE**IPA**INTIASAEPTVHSTP^127–157^ MAIPPKKDQDKTE**IPA**INTIASAEPTVHSTPTTEAVVNAV^127–166^ MAIPPKKDQDKTE**IPA**INTIASAEPTV^127–153^ MAIPPKKDQDKTE**IPA**INTIASAEP^127–151^ MAIPPKKDQDKTE**IPA**INT^127–144^ MAIPPKKDQDKTE**IPA**INTIASAEPTVHSTPTTEAVVNA^127–165^ AIPPKKDQDKTE**IPA**INTIASAEPTVH^128–154^ MAIPPKKDQDKTE**IPA**INTIASAEPTVHSTPTTEAVVNAVDNPE^127–170^ PPKKDQDKTE**IPA**INTIASAEPTVHSTPTTEAVVNAVDNPEA^129–169^ MAIPPKKDQDKTE**IPA**INTIASAEPTVHSTPTTEAVVNAVDNPEA^127–169^ PPKKDQDKTE**IPA**INTIASAEPTV^129–153^ MAIPPKKDQDKTE**IPA**INTIASAEPTVHST^127–156^ MAIPPKKDQDKTE**IPA**INTIASAEPTVHSTPTTEA^127–161^ MAIPPKKDQDKTE**IPA**INTIASAEPTVHSTPTT^127–159^ MAIPPKKDQDKTE**IPA**INTIASAEPTVHSTPTTEAVVN^127–164^ MAIPPKKDQDKTE**IPA**INTIASAEPT^127–152^ MAIPPKKDQDKTE**IPA**INTIASAEPTVHSTPTTEAV^127–162^ MAIPPKKDQDKTE**IPA**IN^127–144^ MAIPPKKDQDKTE**IPA**INTIAS^127–148^ MAIPPKKDQDKTE**IPA**INTIASAEPTVHSTPTTEAVVNAVDN^127–168^ MAIPPKKDQDKTE**IPA**INTIASAEPTVHSTPTTEAVVNAVDNPEASS^127–173^ MAIPPKKDQDKTE**IPA**INTIASAEPTVHSTPTTEAVVNAVDNPEAS^127–172^ MAIPPKKDQDKTE**IPA**INTIASAEPTVHSTPTTE^127–160^ MAIPPKKDQDKTE**IPA**INTIASAEPTVHSTPTTEAVVNAVD^127–167^ MAIPPKKDQDKTE**IPA**INTIASA^127–149^ MAIPPKKDQDKTE**IPA**INTIASAEPTVHSTPT^127–158^ MAIPPKKDQDKTE**IPA**INTIASAE^127–150^ MAIPPKKDQDKTE**IPA**INTIASAEPTVHS^127–155^ MAIPPKKDQDKTE**IPA**INTIASAEPTVH^127–154^	5.40 5.39 5.28 4.69 4.62 4.49 4.46 3.96 3.90 3.84 3.59 3.46 3.37 3.29 3.22 3.14 2.99 2.87 2.78 2.59 2.33 2.31 1.84 1.50 −1.53 −1.71 −1.94 −2.01 −2.18 −2.36 −2.37 −2.39 −2.42 −2.66 −2.77 −2.98 −3.04 −3.16 −3.19 −3.42 −3.52 −3.69 −3.69 −3.73 −3.90 −3.95 −3.96 −4.01 −4.04 −4.11 −4.28 −4.40 −4.64 −4.80 −4.81 −4.82 −5.60 −6.28	DPP-IV inhibitory; Antioxidative;
β-lactoglobulin	VYVEE**LKPTPEG**^59–70^ E**LKPTPEG**NLEILLQ^63–77^ EE**LKPTPEG**NL^62–72^ VYVEE**LKPTPEG**NL^59–72^ VYVEE**LKPTPEG**NLE^59–73^ EE**LKPTPEG**NLEILL^62–76^	2.10 2.03 1.99 1.74 1.69 1.67	DPP-IV inhibitory; Antioxidative;
β-casein	EMPFPK**YPVEPF**T^122–135^ MPFPK**YPVEPF**TE^123–136^ EMPFPK**YPVEPF**TE^122–136^ MPFPK**YPVEPF**T^123–135^ MPFPK**YPVEPF**TES^123–137^ EMPFPK**YPVEPF**TES^122–137^ MPFPK**YPVEPF**^123–134^ EMPFPK**YPVEPF**^122–134^ VKETMVPKHKEMPFPK**YPVEPF**TESQSLTLTDVE^113–156^ HKEMPFPK**YPVEPF**TESQ^121–138^ HKEMPFPK**YPVEPF**TESQSLTLTDVEKLH^121–149^ HKEMPFPK**YPVEPF**TESQSLT^121–141^ HKEMPFPK**YPVEPF**TESQSLTLTDVE^121–146^ HKEMPFPK**YPVEPF**TESQSLTLTDVEKLHLPLPLVQ^121–156^ HKEMPFPK**YPVEPF**TESQS^121–138^ HKEMPFPK**YPVEPF**TESQSL^121–139^ VKETMVPKHKEMPFPK**YPVEPF**TESQSL^113–140^ HKEMPFPK**YPVEPF**TESQSLTLTDVEK^121–147^ VKETMVPKHKEMPFPK**YPVEPF**TESQS^113–139^ EMPFPK**YPVEPF**TESQSLTLTDVEKLHLPLP^122–153^ HKEMPFPK**YPVEPF**TESQSLTLTDVEKLHLPLP^121–153^	4.23 4.01 3.96 3.90 3.39 3.15 1.93 1.90 −1.50 −1.57 −1.59 −1.64 −1.73 −1.74 −1.75 −1.90 −2.55 −2.66 −2.67 −2.75 −3.66	DPP-IV inhibitory; Antibacterial;
β-casein	**YQEPVLGPVR**^206–215^ **YQEPVLGPVR**GPFP^206–219^ VLPVPQKAVPQRDMPIQAFLL**YQEPVLGPVR**GPFP^185–219^ AVPQRDMPIQAFLL**YQEPVLGPVR**GPFPI^192–220^ SLSQPKVLPVPQKAVPQRDMPIQAFLL**YQEPVLGPVR**GPFPILV^178–222^ AVPQRDMPIQAFLL**YQEPVLGPVR**GPFP^192–219^ FLL**YQEPVLGPVR**GPFP^203–219^ VLPVPQKAVPQRDMPIQAFLL**YQEPVLGPVR**GPFPILV^185–222^ VLPVPQKAVPQRDMPIQAFLL**YQEPVLGPVR**GPFPI^185–220^ **YQEPVLGPVR**GPFPIIV^206–222^ **YQEPVLGPVR**GPFPILV^206–222^ VLPVPQKAVPQRDMPIQAFLL**YQEPVLGPVR**GPFPIL^185–220^	2.10 −1.61 −1.64 −1.75 −1.77 −1.80 −2.10 −2.14 −2.24 −2.31 −2.31 −2.33	DPP-IV inhibitory; Antioxidative;
β-lactoglobulin	**VYVEEL**KPTPEG^59–70^ **VYVEEL**KPTPEGNL^59–72^ **VYVEEL**KPTPEGNLE^59–73^	2.10 1.74 1.69	DPP-IV inhibitory; Antibacterial; Antioxidative;
β-lactoglobulin	ENKVLV**LDTDYKK**Y^107–120^ VLV**LDTDYKK**Y^110–120^ **LDTDYKK**YL^113–121^ VLV**LDTDYKK**^110–119^ IDALNENKVLV**LDTDYKK**Y^102–120^ LV**LDTDYKK**Y^111–120^ VLV**LDTDYKK**YL^110–121^ V**LDTDYKK**Y^112–120^ LV**LDTDYKK**YL^111–121^ **LDTDYKK**YLL^113–122^ ENKVLV**LDTDYKK**^107–119^ DALNENKVLV**LDTDYKK**Y^103–120^ **LDTDYKK**Y^113–120^ IDALNENKVLV**LDTDYKK**^102–119^ LV**LDTDYKK**^111–119^ ENKVLV**LDTDYKK**YL^107–121^ VLV**LDTDYKK**YLL^109–122^ DALNENKVLV**LDTDYKK**^103–119^ KVLV**LDTDYKK**Y^109–120^	3.45 3.42 3.18 3.05 3.05 2.94 2.72 2.62 2.52 2.48 2.48 2.43 2.38 2.33 2.23 2.18 2.17 1.90 1.67	DPP-IV inhibitory; Antibacterial; Antioxidative;
β-lactoglobulin	IPAVFK**IDALNENK**^106–109^ **IDALNENK**VLVLDTDYKKY^102–120^ **IDALNENK**VL^102–111^ TKIPAVFK**IDALNENK**^94–109^ **IDALNENK**VLVLDTDYKK^102–119^ KIPAVFK**IDALNENK**^95–109^ K**IDALNENK**^101–109^ VFK**IDALNENK**^99–109^ **IDALNENK**V^102–110^	3.09 3.05 2.60 2.46 2.33 2.30 2.21 1.99 1.96	DPP-IV inhibitory; Antioxidative;
β-lactoglobulin	IPAVFK**IDALNENK**^106–109^ **IDALNENK**VLVLDTDYKKY^102–120^ **IDALNENK**VL^102–111^ TKIPAVFK**IDALNENK**^94–109^ **IDALNENK**VLVLDTDYKK^102–119^ KIPAVFK**IDALNENK**^95–109^ K**IDALNENK**^101–109^ VFK**IDALNENK**^99–109^ **IDALNENK**V^102–110^	3.09 3.05 2.60 2.46 2.33 2.30 2.21 1.99 1.96	DPP-IV inhibitory; Antioxidative;
β-lactoglobulin	IPAVFK**IDALNENK**^106–109^ **IDALNENK**VLVLDTDYKKY^102–120^ **IDALNENK**VL^102–111^ TKIPAVFK**IDALNENK**^94–109^ **IDALNENK**VLVLDTDYKK^102–119^ KIPAVFK**IDALNENK**^95–109^ K**IDALNENK**^101–109^ VFK**IDALNENK**^99–109^ **IDALNENK**V^102–110^	3.09 3.05 2.60 2.46 2.33 2.30 2.21 1.99 1.96	DPP-IV inhibitory; Antioxidative;
β-lactoglobulin	LDIQ**KVAGT**WHS^28–39^ IIVTQTMKGLDIQ**KVAGT**WH^19–38^	1.92 −2.06	DPP-IV inhibitory; Antioxidative;
β-lactoglobulin	**AASDISLLDAQSAPLR**^43–58^ LAMA**ASDISLLDAQSAPLR**^40–58^ M**AASDISLLDAQSAPLR**^42–58^ **AASDISLLDAQSAPLR**V^43–59^	2.57 2.37 1.88 1.53	DPP-IV inhibitory; Antibacterial;
β-lactoglobulin	TPEVDNEALEKFDK**ALK**^143–159^ TPEVDNEALEKFDK**ALK**A^143–160^ DNEALEKFDK**ALK**^147–159^ EVDNEALEKFDK**ALK**^145–159^ NEALEKFDK**ALK**^148–159^ EALEKFDK**ALK**ALPMH^149–164^ NEALEKFDK**ALK**ALPMH^148–164^ NEALEKFDK**ALK**ALPMHIR^148–166^ EALEKFDK**ALK**ALPMHIR^149–166^	4.03 2.92 2.38 2.01 −1.62 −1.77 −2.37 −2.65 −2.96	DPP-IV inhibitory; Antioxidative;

The active sequences contained in longer peptides are highlighted with bold characters.

## 4. Conclusions

Due to its higher content of nutrients compared to other species, ovine scotta is a precious substrate that can be valorized through a multidisciplinary biotechnological approach with the aim of producing ingredients with specific biological activities. The enzymatic hydrolyses performed both with bromelain and pancreatin on retentate of scotta allowed enhancement of its DPP-IV inhibitory and antioxidant activities, bromelain being more promising in such an aim. Likewise, the antibacterial activity of hydrolysates slightly increased with respect to control, even if an inhibitory effect against some Listeria monocytogenes strains of the non-hydrolysates scotta was also noticed. LC-MS/MS analysis allowed identification among the experimental groups of several differential peptides that contain sequences with known activities among those here studied. Further studies are needed to optimize reaction conditions, in order to maximize such biological activities in relation to the specific objective.

## Figures and Tables

**Table 1:** List of microorganisms, medium and culture condition for testing the antimicrobial activity of enzymatic hydrolysate of Scotta ^1^.

Tested Organisms	Source	Medium	Temperature and Time of Incubation
*Staphylococcus aureus* 20,231 DSMZ	DSMZ	BHI	37 °C × 24 h
*Listeria monocytogenes* B *Listeria monocytogenes* C	DAFS	BHI	37 °C × 24 h
*Listeria monocytogenes* E	DAFS	BHI	37 °C × 24 h
*Listeria monocytogenes* 20,600 DSMZ	DSMZ	BHI	37 °C × 24 h
*Salmonella bongori* 13,772 DSMZ	DSMZ	BHI	37 °C × 24 h

^1^ DSMZ, Deutsche SammLung von Mikroorganismen und Zellkulturen, German Collection of Microorganism of Cell Cultures; DAFES, Collection of Microorganisms of Dipartimento di Agraria of the University of Sassari, Section of Food and Environmental Science.

**Table 2:** DPP-IV and antioxidant activity of hydrolysates, and control ^1^.

Run	BSPH	PSPH	CTRL
DPP-IV IC_50_ (mg mL^−1^)	8.5 ^b^ ± 0.2	13 ^a^ ± 1	n.d.
ABTS IC_50_ (mg mL^−1^)	0.79 ^b^ ± 0.03	0.87 ^ab^ ± 0.01	1.06 ^a^ ± 0.18

^1^ Values are mean ± standard deviation (*n* = 3). Within rows, values with the same letter do not differ significantly from each other according to LSD test (*p* < 0.05). n.d.: absence of inhibition.

## Data Availability

The peptides identified in this study were analyzed for potential activity with the tool available on BIOPEP-UWM database at the following link: https://biochemia.uwm.edu.pl/en/biopep-uwm-2/.

## Supplementary Materials

The following are available online at https://www.mdpi.com/article/10.3390/foods10123137/s1, Sheet S1: All identified peptides in BSPH, PSHP, and CTRL. Sheet S2: Differential proteins analysis of BSPH vs. CTRL. Sheet S3: Differential peptides analysis of BSPH vs. CTRL. Sheet S4: Differential proteins analysis of PSPH vs. CTRL. Sheet S5: Differential peptides analysis of PSPH vs. CTRL. Sheet S6: Differential proteins analysis of BSPH vs. PSPH. Sheet S7: Differential peptides analysis of BSPH vs. PSPH.
